# Forensic taphonomic experimental design matters: a study assessing clothing and carrion biomass load on scavenging in Cape Town, South Africa

**DOI:** 10.1007/s00414-024-03171-w

**Published:** 2024-02-19

**Authors:** Kara Sierra Adams, Devin Alexander Finaughty, Victoria Elaine Gibbon

**Affiliations:** 1 Division of Clinical Anatomy and Biological Anthropology, Department of Human Biology, Anatomy Building, Level 5, Room 5.14, Faculty of Health Sciences, University of Cape Town, , Anzio Rd, Observatory, Cape Town, 7925 South Africa; 2 School of Chemistry and Forensic Science, Division of Natural Sciences, University of Kent, Canterbury, Kent UK

**Keywords:** Post-mortem interval, Taphonomy, Scavenging, Western Cape, Decomposition, Ecology, Peri-urban environment, Open Cape Flats Dune Strandveld, Biogeographical region, Carrion load, Seasonality

## Abstract

The identification of unknown human remains is a significant and ongoing challenge in South Africa, worsened by the country’s high murder rate. The rate of decomposition in South Africa is significantly influenced by vertebrate scavenging, which, if not considered, can impede the accurate estimation of the post-mortem interval. Scavenging patterns vary greatly depending on the environment and ecological region, and there is limited data for the Western Cape province. To address this gap, two clothed and uncaged pig carcasses weighing 60 kg each were placed in the field in July 2021 and January 2022, respectively. Motion-activated infrared-capable trail cameras were used to observe decomposition, scavenger species, and their activities. Additionally, a comparative sample of 16 unclothed carcasses deployed between 2014 and 2016 in the same habitat were analyzed to assess the impact of clothing and biomass load. The study found three main results: (1) Regardless of habitat or biomass load, it took significantly less time to reach decomposition milestones (25%, 50%, and 75%) during the summer season; (2) the presence of mongoose scavengers had a greater impact on the time required to reach milestones during winter compared to summer; and (3) single carcass deployments reached the milestones faster than multi-carcass deployments in both seasons. This research highlights the potential inaccuracy of current methods for estimating the post-mortem interval when scavenging activity is not considered or documented in the underlying experimental data, particularly for environments or ecological biomes where scavengers actively impact decomposition rates.

## Introduction

An accurate post-mortem interval (PMI) estimate is critical for identification of unknown decedents in medico-legal death investigations as it can narrow the pool of potential victims and limit the number of potential perpetrators by providing a timeframe when the person died [1]. It relies on an accurate analysis of the body’s taphonomic state with an understanding of the numerous local intrinsic and extrinsic taphonomic factors that affect the decomposition process [2–5]. Therefore, it is necessary to identify and document the specific role different factors have on the taphonomic process in the multitudinous circumstances a body may be found. Amongst the most impactful factors is scavenging by vertebrates and invertebrates. Environmental impacts (region, weather, season), the body (carcass size, condition, length of exposure), and the present scavenger species, all influence scavenging patterns and, by extension, the rate and pattern of decay [6–10]. The need for understanding and quantifying the interactions between these variables is particularly relevant in South Africa given the country’s high number of unidentified persons. Around 9–10% of bodies entering South African mortuaries are unclaimed resulting in thousands of unidentified persons per annum [11–16]. Data from Forensic Anthropology Cape Town which serves the Western Cape region states that most individuals were skeletonised (70%; 121/174), followed by those in an advanced state of decomposition (19%; 33/174) [1]. An aspect of this is the high murder rate in South Africa 2022/2023 there were 27,494 murders, with 4150 coming from the Western Cape Province where the present research is sited [17]. One of the centres for crime in the Western Cape is the City of Cape Town, notably the Cape Flats portion, which extends from the Boland Mountains in the east across to Table Mountain range in the west. This forensically significant region is characterized by a dense population (> 13,000 inhabitants per square kilometre) [18] and high levels of interpersonal violence, including murder. The suburb where the present research is located, Delft, reported 277 murders in 2022/2023 alone [17]. Local taphonomic research conducted in this area by Finaughty [19] identified the Cape grey mongoose (*Galerella pulverulenta*) as a primary scavenger within the Cape Flats Dune Strandveld (CFDS) habitat, which characterizes much of the Cape Flats region. The impact of mongoose scavenging on the decomposition process was further investigated by Spies et al. [20, 21] where scavenged and un-scavenged porcine carcasses were compared. Further research was conducted to understand the effect clothing has on scavenging behaviour and the taphonomic process in a thicketed environment within the CFDS habitat [20, 21]. The results noted significant differences regarding the decomposition of clothed and unclothed bodies as well as the significance of the Cape grey mongoose as an active taphonomic agent. This research highlighted the importance for conducting forensically realistic experimental field work that employs single, clothed, uncaged carcasses [20, 21].

Scavenging is a significant taphonomic factor to study as it is well established to alter decomposition rate and patterns through consumption of carrion tissue and/or invertebrate scavengers, distribute and scatter carcass elements, and mimic peri- and post-mortem anthropogenic interaction with the remains [9, 22–26]. Numerous experimental studies to investigate the role of local scavengers in the decomposition process have been completed, but scavenger guilds are biogeographically specific [27–29]. South Africa comprises multiple biogeographic zones, each with unique microenvironments, presenting with their own set of taphonomic factors impacting scavenger guilds. Thus, the accuracy of estimating PMI for forensic anthropological medico-legal death investigations in each of these locales hinges on having knowledge of the local scavenger guild and its interaction with the decomposition ecosystem.

While local baseline data exist for scavenging in the thicketed CFDS environment, there is a lack of detailed understanding for the effects of experimental design on scavenging activity generally, and for this biogeographic region specifically. Thus, the aim of the present research is to identify the relevant scavenging species in the forensically significant, peri-urban open CFDS habitat in Cape Town, South Africa. The study objectives include analyzing and categorizing scavenging behaviours of animals identified to interact with carcasses and to analyze the impact of clothing and carrion biomass load on scavenging activity across seasons in the open CFDS. As most (39%) forensic anthropology medico-legal death investigations analyzed from Forensic Anthropology Cape Town (FACT) were clothed [24, 25], this research aims to provide locally relevant baseline data.

## Materials and methods

**Fig. 1:**
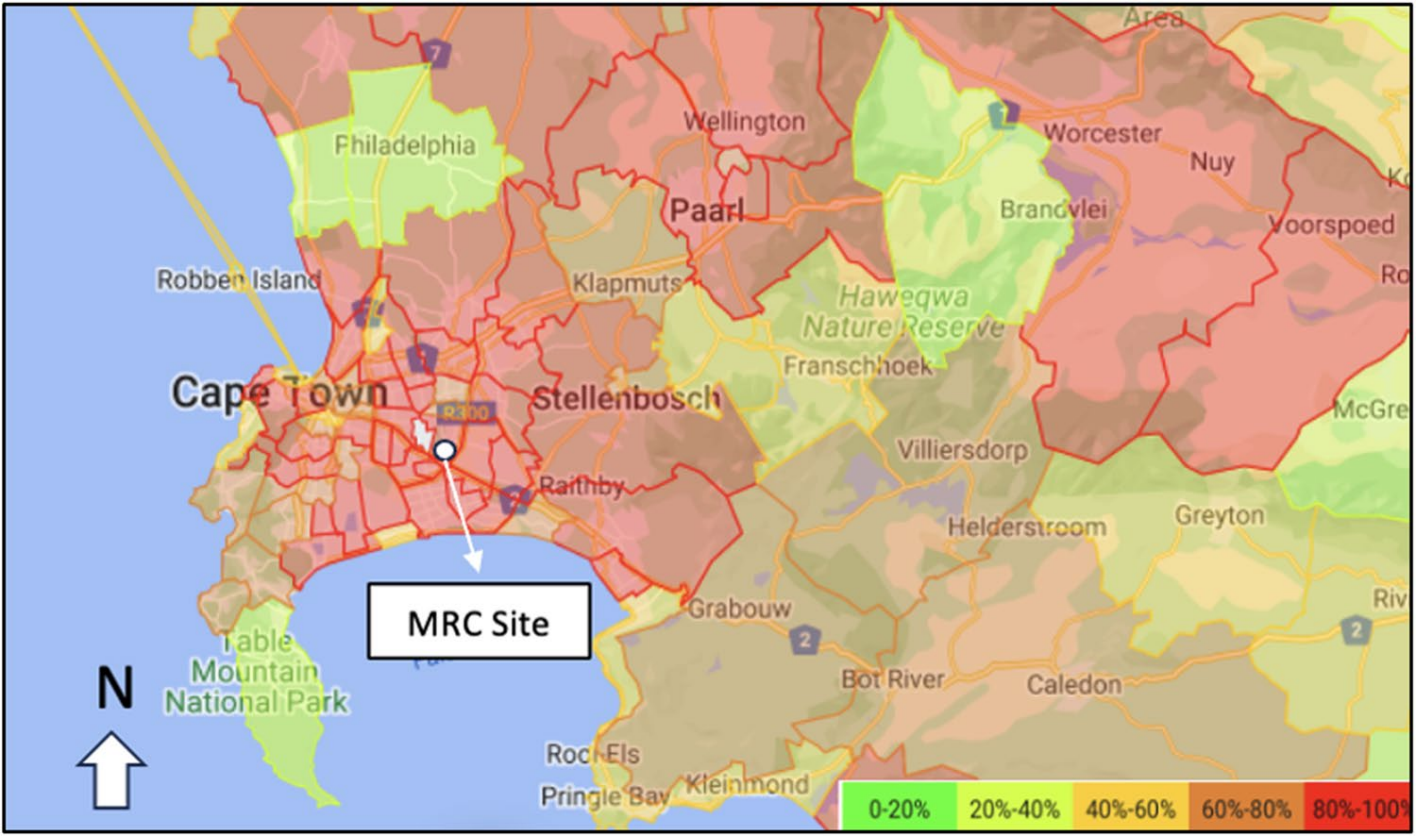
Murder rate by policing district with research location indicated on the original map Crime Statistics South Africa. The Cape Flats region’s policing precincts are heat-mapped to show the quintiles of contact crimes (murders), where precincts in the fifth quintile (top 20%) in terms of the numbers of murders nationally are indicated in red (legend in http://www.crimestatssa.com)

The research site was located at the South African Medical Research Council’s research facility (MRC), in Delft, Cape Town, South Africa (Fig. 1). This region of South Africa is characterized by a Mediterranean climate, with cold wet winters and hot, dry summers (Csb—Köppen-Geiger climate classification). The site is an outdoor peri-urban environment (UTM 17 T 283009.88 m E, 6,236,344.50 m S). The two habitat types are classed as “CFDS open” and “CFDS thicketed” (or variants thereof), respectively.

**Fig. 2:**
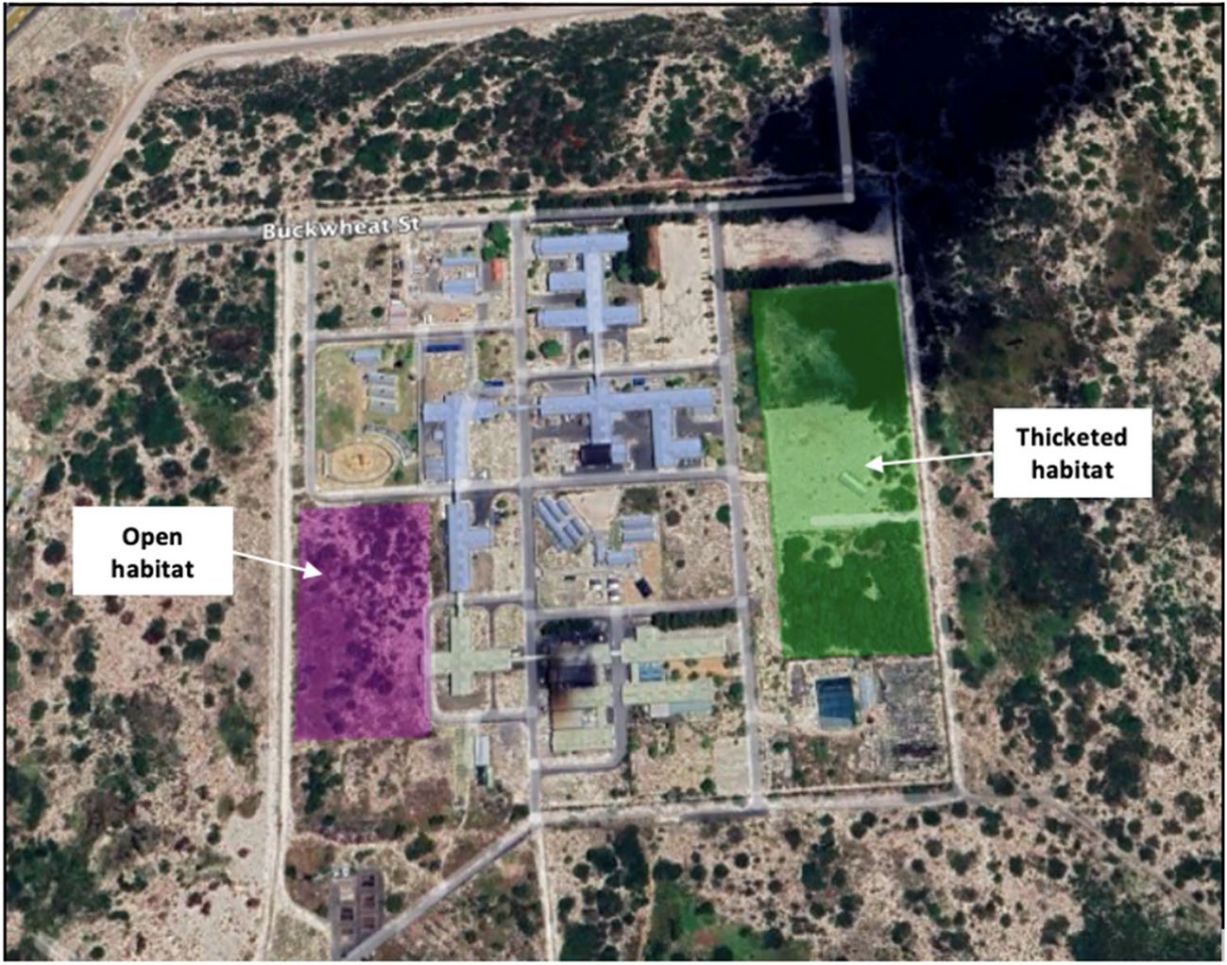
MRC thicketed (green) and open (pink) research sites located in Delft indicated on the map using google earth

The CFDS open site is 3.64 acres and located on the west side of the MRC facility. The entire facility is enclosed by a 6-foot electric fence overlying sandy soil, easily accessed by small and medium-sized animals who can fit/dig underneath the fence but is designed to deter access by people and livestock. The CFDS thicketed site is approximately 6 acres and located on the east side of the MRC facility, with characteristic dense thickets of invasive tree species, Port Jackson (*Acacia saligna*) and Rooikrans (*Acacia cyclops*). The urban settlements of Delft have encroached up to the MRC fence, but the site was adjacent to the Driftsands Nature Reserve, which is one of the largest remaining intact remnants of CFDS vegetation. A large portion of medico-legal death investigations come from this vegetation type given its ubiquitous coverage across a range of forensically active regions of the Cape Flats. CFDS is characterized by flat to slightly undulating landscapes with sandy, nutrient poor soils [30]. Though previously ploughed for agricultural purposes, the land at the research site has reverted to its natural state [19]. Strandveld vegetation is characterized by tall evergreen, hard leaved shrubland with abundant grasses, annual herbs with succulents in the gaps [30]. However, because of the previous ploughing, there has been an invasion of Port Jackson (*Acacia saligna*) and Rooikrans (*Acacia cyclops*) bushy trees, which occur in small pockets through the open CFDS, but not nearly to the same degree of coverage as the thicketed site, as evident in the satellite images in Fig. 2.

### Sample

Six 60-kg pig (*Sus scrofa domesticus*) carcasses were used as analogues for adult human bodies. The use of pigs as proxies for humans in taphonomic studies where the establishment of baseline data are concerned is widely accepted where the use of humans is prohibited [31, 32], and most taphonomic studies regularly use porcine carcasses for taphonomic research. The six porcine carcasses were deployed on July 1st, 2021, and January 13th, 2022. They were terminated by a single 0.22 calibre gunshot wound to the base of the brain. To ensure the animals’ welfare, this process was carried out by an industry professional, per the ethically approved protocol (FHS AEC 018_023). Following termination, within an hour of death, the carcasses were washed with water, and placed into body bags. Following the placement of the carcasses in body bags, the bodies were transported immediately to the two research sites without any refrigeration or freezing. In keeping with forensic realism, the carcasses were deployed within two and a half hours of termination and clothed on site. Previous analysis of Forensic Anthropology Cape Town (FACT) case files conducted by Spies [33] provided details regarding the most common clothing types. The clothing used included the following: underwear, cotton T-shirt, denim pants, and a leather belt in the summer and the addition of socks, shoes, and jerseys in the winter [33, 34]. To ensure the accurate fit of clothing, alterations were made to accommodate the anatomical differences between humans and pigs. Spies [34] used measurements taken from a live 60-kg pig as a guide for tailoring the clothes: chest circumference was 87 cm; pelvis circumference was 81 cm; armpit height was 31 cm; shoulder height was 54 cm; groin height was 33 cm; thigh circumference was 45 cm; snout-to-tail length was 136 cm. Clothes were purchased in sizes to reflect these measurements. After alterations were made including shortening and tapering the pant legs and jersey arms according to the measurements detailed above.

To assess the effect of clothing and biomass load on scavenging, a dataset consisting of 16 unclothed ~ 60-kg pig carcasses (2014–2016) were included; eight deployed in the same CFDS open habitat and eight in the CFDS thicketed habitat [19]. The dataset consisted of recordings of daily weight loss, multiple weather variables, and scavenging activity.

### Carcass decomposition

Decomposition rate (by proxy of carcass weight loss in kilograms over time) was determined using a solar-powered automated, remotely accessible weighing apparatus [35]. Once every 24-h period (at midnight), the scale would lift for 10 s, weight readings would be taken, and each lift generated an email that was sent to the researcher listing the weight of the carcass. Twenty readings were obtained during each lift and the average was used. Decomposition was visually assessed from photos obtained once per day from a standardized position directly over the carcass (imaging apparatus described in next section). This was accomplished using the visual criteria developed by Keough et al*.* [36] for the head and neck, and abdomen only, as clothing prevented visibility of the limbs. The visual scoring was only used to assess scavenging behaviour and activity of the single carcass deployments. Scoring based on visual observations was omitted in the comparison of single carcass versus multi-carcass deployments. The preference for weight loss over time was adopted as a more standardized approach for quantifying decomposition, particularly given variations such as the presence of clothing on certain carcasses.

### Scavenging activity

Scavenging was constantly monitored throughout each deployment by a motion-activated, infrared-capable time-lapse wildlife trail camera (Foxelli Oaks Eye Trail Camera). The wide-angled camera was mounted above the carcass and was programmed to capture one photo at each hourly interval. An additional motion activated trail camera (Primos Proof Cam) a few metres away pointed directly towards the carcasses was programmed to take three photos when triggered, rearming after 60 s. All camera trap data were analyzed to document seasonal scavenging patterns using TimeLapse 2. Scavenging behaviour was categorized based on an adaptation of the criteria described by Dibner et al. [37]. All activity was categorized in one of three categories: “no contact” (the animal was visible but not on the grid of the rig); “close observation” (the animal was touching the weigh grid, but clearly not touching the carcass); and “feeding/ direct contact” (the scavenger was either in direct contact with the carcass or had remains visibly in its mouth). Visits were defined by the absence of the scavenger for 10 min or longer as described in previous research [19, 33, 38].

## Results

Two single clothed carcasses were deployed in winter 2021 and summer 2022 in the CFDS open habitat, referred to as OSC (open single clothed) from here on. Sixteen unclothed carcasses were deployed—four at a time—between 2014 and 2016 in both the CFDS open and CFDS thicketed habitats, referred to as OMU (open multi unclothed) and TMU (thicketed multi unclothed), respectively, from here on. The following scavenging and decomposition data were recorded and are presented below.

To assess the effect of seasonality as a confounding factor in the decomposition process, weather data from the current experiments and the comparative dataset were compared. The specific weather variables and values are available in Table 1 along with any significant differences between deployments and season, as explained below.

**Table 1:**
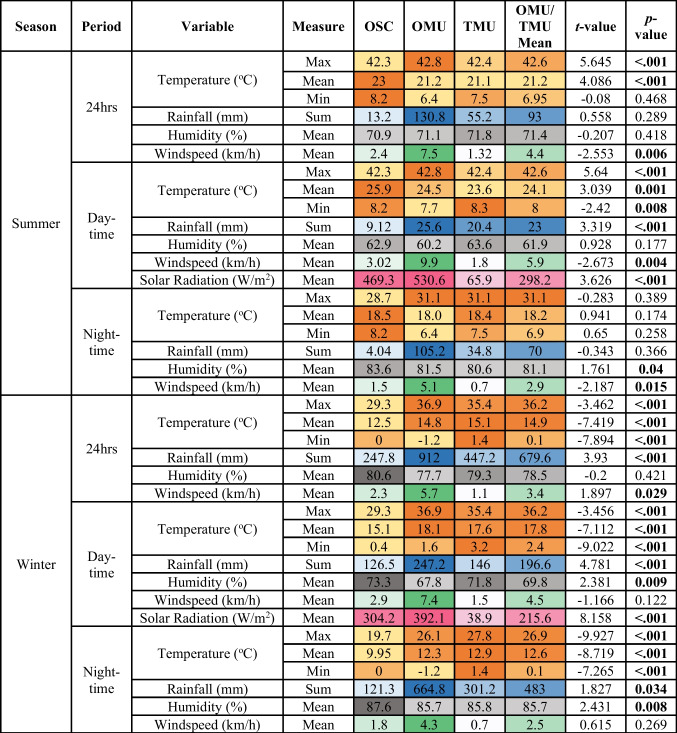
Average weather variables recorded during all four cycles for 24 h, daytime, and night-time, along with deployment differences (values are heat-mapped to indicate intensities/proportions; different colours are used for the different variables, and lighter colours indicate lower values, while deeper colours are higher values); *OSC* clothed open single carcass data, *TMU* unclothed thicketed multi-carcass data, *OMU* unclothed open multi-carcass data, *°C* degrees Celsius, *mm* millimetres, *%* percentage, *W/m*^*2*^, Watts per square metre, *km/h* kilometres per hour; *t*-statistic from Welch two-sample *t*-test; significant differences (*p* < 0.05) are bolded

In winter, differences in temperature between the OSC deployment and Finaughty’s [19] deployments were found to be significantly different, with the OSC deployment having a cooler mean temperature. Windspeed and rain were also significantly different and Finaughty’s deployments experienced mean wind speeds of 3.4 km/h, with the OMU carcasses experiencing 4.6 km/h greater windspeeds than the TMU carcasses, while the OSC deployments experienced speeds of 2.3 km/h. It rained significantly more during Finaughty’s deployments, the mean TMU and OMU deployments receiving 432 mm more rain than the OSC deployment. Additionally, the difference in solar radiation was found to be significantly different, with Finaughty’s deployments having a mean value of 215.6 W/m^2^, and the present study’s deployments experiencing a value of 304.2 W/m^2^; the TMU carcasses had the lowest solar radiation with a mean value of 38.9 W/m^2^, explained by the heavy vegetative cover overlying the carcasses.

In summer, differences in temperature between the single carcass deployments and Finaughty’s [19] deployments were found to be significantly different, except for the minimum daytime temperature and maximum nighttime temperature. Finaughty’s deployments experienced greater summer rainfall with a mean of 93 mm of rainfall while the OSC deployment received 13.4 mm of rain. Windspeed differences were determined to be statistically significant, with Finaughty’s deployments experiencing average wind speeds of 4.4 km/h while Adams’s deployments experienced average wind speeds of 1.7 km/h. Finally, solar radiation was significantly different between the deployments. The OSC deployment had a mean solar radiation value of 425.3 W/m^2^, while Finaughty’s had a mean solar value of only 298.2 W/m^2^.

### Scavenging behaviour and activity

**Fig. 3:**
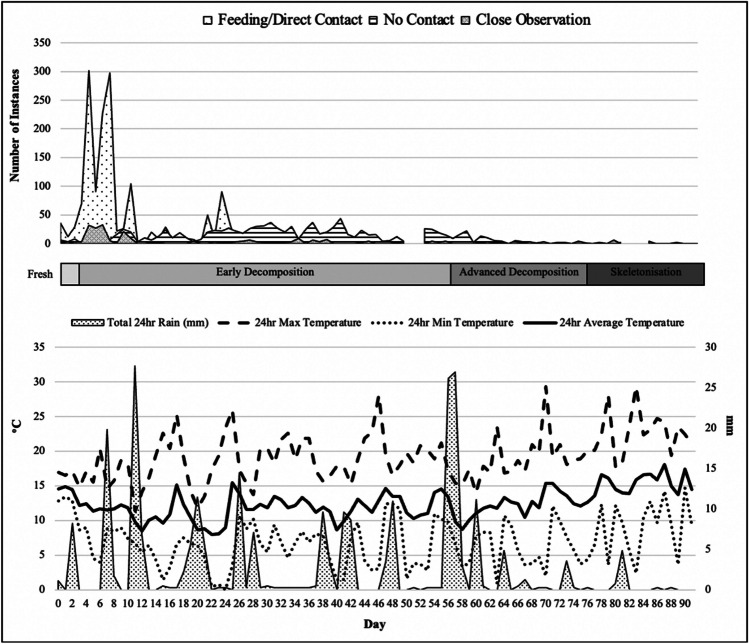
Cape grey mongoose scavenging behaviour recorded in the open habitat at the Medical Research Council’s research facility in Delft, Cape Town for winter 2021. Weather data for same period presented below scavenging activity. °C, degrees Celsius; mm, millimetres

**Fig. 4:**
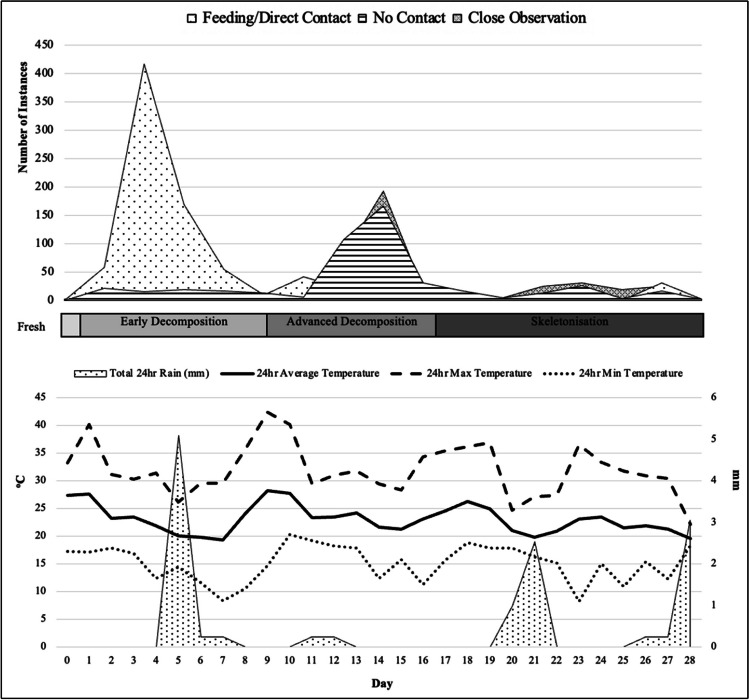
Cape grey mongoose scavenging behaviour recorded in the open habitat at the Medical Research Council’s research facility in Delft, Cape Town for summer 2022. Weather data for same period presented below scavenging activity. °C, degrees Celsius; mm, millimetres

The Cape grey mongoose (*Galerella* pulverulenta) was the only vertebrate scavenger documented to feed on the remains. A summary of the scavenger behaviours from both deployments can be observed in Table 2 and Figs. 3 and 4 stacked area plots. In both current experimental deployments, feeding and direct contact by the Cape grey mongoose largely occurred during the fresh and early stages of decomposition. Winter feeding occurred from Day 0 until Day 70, while in the summer feeding occurred from Day 0 until Day 17, though feeding did not occur every day in either of the deployments. Following the initial feeding exhibited by the mongoose in the first few days post-deployment, their scavenging behaviour was found to differ between seasons, with the winter season feeding presenting in a bimodal pattern, peaking in the fresh/beginning of early decomposition phase and then again during middle early decomposition. In comparison, summer feeding peaked only during the early decomposition phase. Both seasons showed a reduction in feeding activity at advanced decomposition onset. An interesting behaviour witnessed during summer was the peak in close observation/no contact behaviour, which occurred intensely for 4 days during advanced decomposition; the mongoose dug under the grid, a behaviour undocumented during winter. In winter, the mongoose spent extra time around the carcass not feeding, but cleaning, resting, and observing; none of these activities were documented during the summer season.

**Table 2:** Scavenging behaviours of the Cape grey mongoose recorded in the open habitat at the medical research council facility in Delft, Cape Town, compared between seasons

Season	Carcass ID	Measure	Total visits	Feeding/direct contact	Close observation	No contact	Multi- mongoose visits
Winter	OSC	Count	351	1633	244	1015	20
		Duration (hh:mm:ss)	21:04:19	09:07:40	02:23:13	9:33:17	00:47:04
Summer	OSC	Count	135	825	448	472	7
		Duration (hh:mm:ss)	14:19:55	6:31:18	2:37:41	5:10:56	00:27:34

**Fig. 5:**
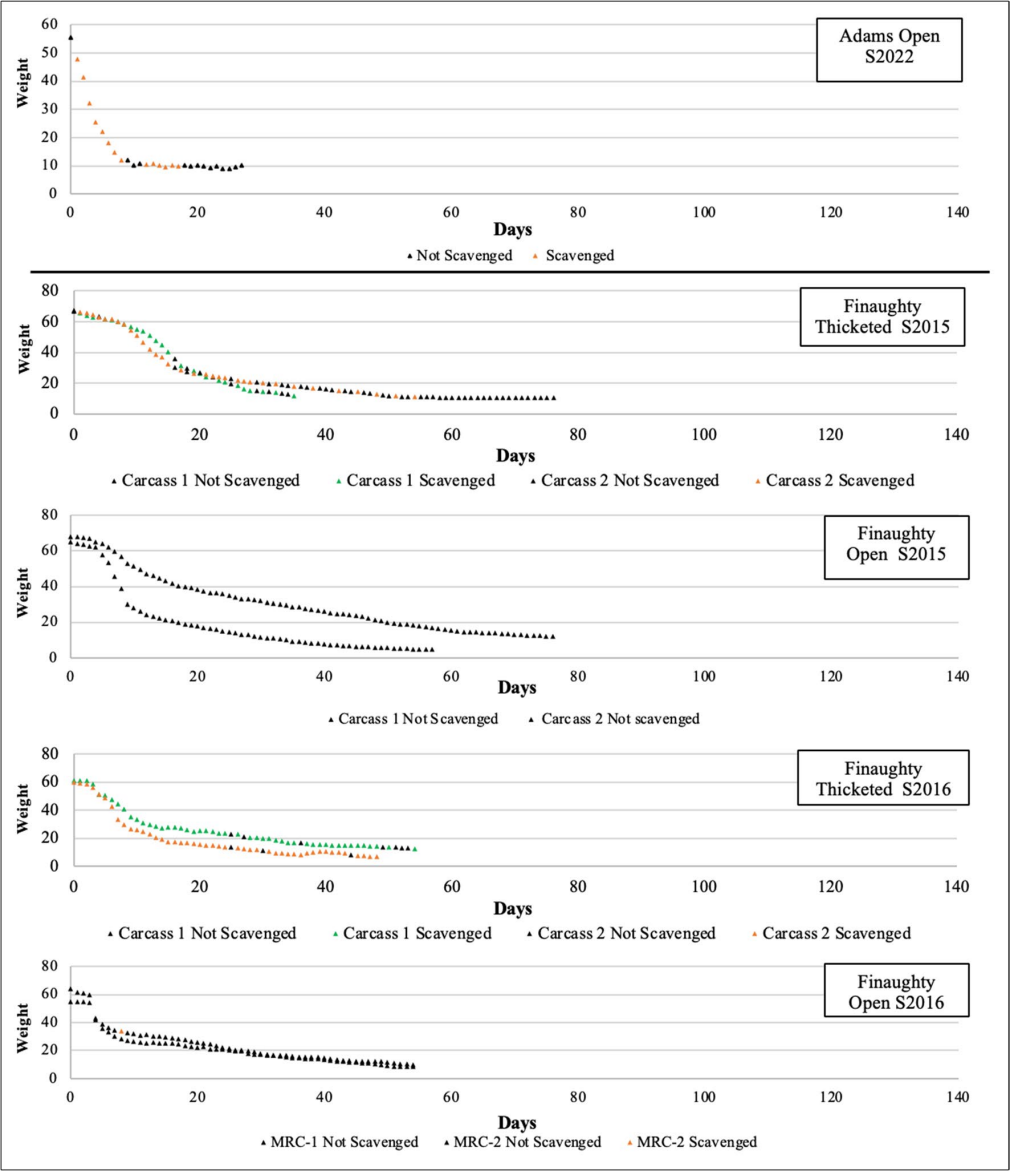
The presence and absence of scavenging activity recorded in closed and open habitats at the Medical Research Council’s research facility in Delft, Cape Town, across all winter deployments from 2014 to 2016 thicketed multi-carcass unclothed data (TMU) and the open multi-carcass unclothed data (OMU), and 2021–2022 open single clothed carcass data (OSC)

**Fig. 6:**
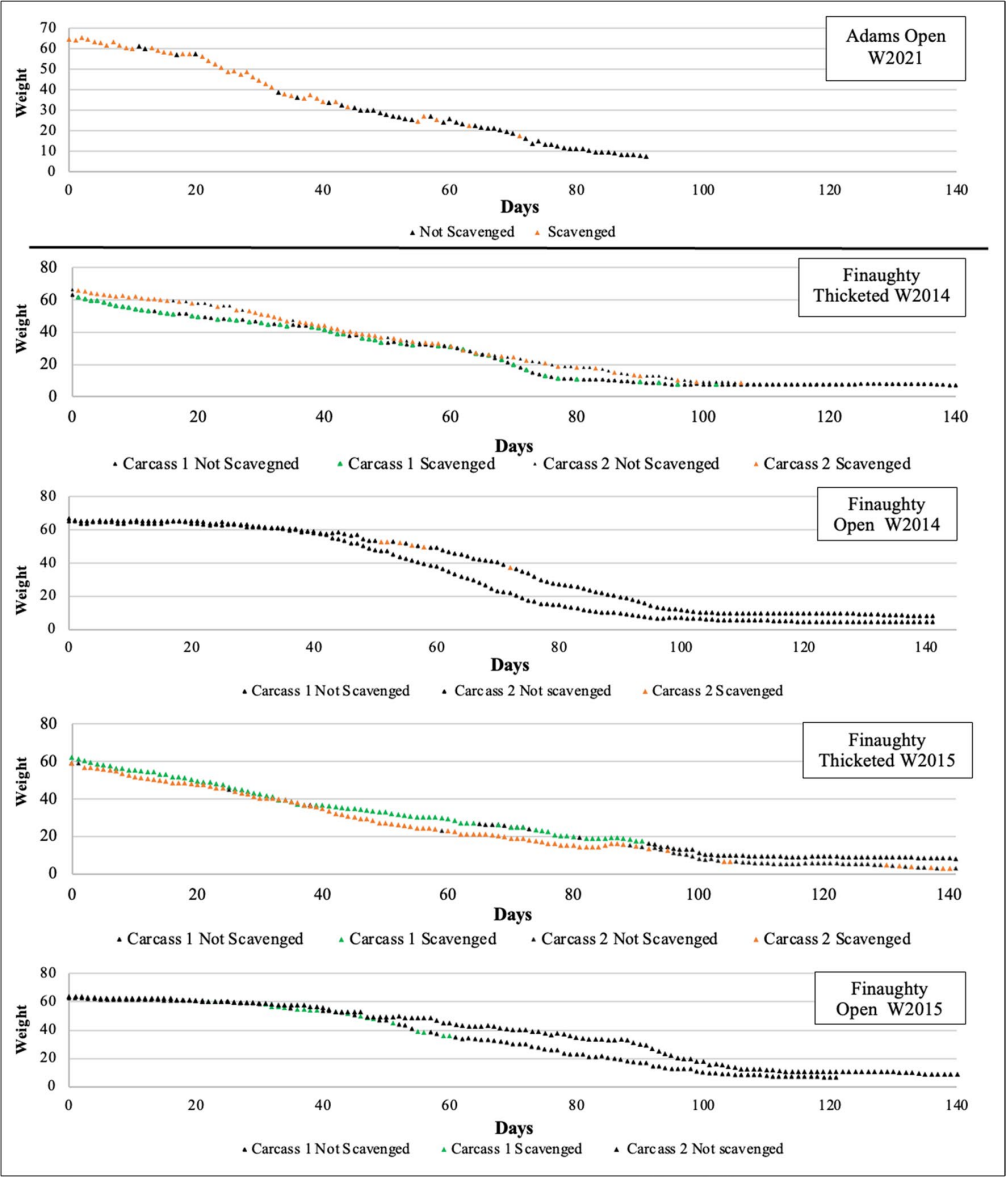
The presence and absence of scavenging activity recorded in closed and open habitats at the Medical Research Council’s research facility in Delft, Cape Town, across all summer deployments from 2014 to 2016 unclothed thicketed multi-carcass data (TMU) and the unclothed open unclothed multi-carcass data (OMU), and 2021–2022 clothed single carcass data (OSC)

Between the 2014 and 2016 open habitat deployments, scavenging data were not recorded in detail. The available data were translated into a presence/absence of daily scavenging for all the deployments. The presence/absence of scavenging are presented Figs. 5 and 6. The OSC carcasses were heavily scavenged in both summer and winter. Comparatively, the OMU carcasses in the same habitat were very rarely or never scavenged while the TMU carcasses experienced heavy scavenging. However, when the data were compared with the single carcass deployments that occurred between 2021 and 2022, the pattern was most like the carcasses deployed in the thicketed area. Both single carcass deployments experienced a similar number of feeding days (46% in winter and 52% in summer) as the multi carcass deployments in the thicketed habitat (59% in the winter and 72% in the summer). In comparison, the carcasses in the open habitat during the multi-carcass deployments experienced less, around 5% of days spent scavenging during winter and less than 1% of the days in summer.

A comparison of scavenging activity at the clothed and unclothed carcasses is presented in Table 3. The clothed and unclothed carcasses showed similar visit times by day in summer and winter, but overall visit number over the deployment was greater in the unclothed carcasses. The TMU carcasses experienced 673 documented mongoose visits in winter and 399 in the summer. Comparatively, the OSC carcasses experienced 351 documented visits in winter and 135 in summer; winter receiving nearly double and summer three times the number of visits. Regarding total visit duration, the unclothed carcass average total time was five times greater at 111:30:05 compared to 21:04:19 for winter. This same pattern was observed in summer with the multiple carcasses visited for a mean time of 71:27:52 compared to 14:19:55.

**Table 3:** Number of visits and visit duration of the Cape grey mongoose for clothed and unclothed carcasses deployed in the CFDS open and CFDS thicketed habitats at the Medical Research Council’s research facility in Delft Cape Town, for winter and summer seasons. *OSC* clothed open single carcass data, *TMU* unclothed thicketed multi-carcass data

Clothing	Season	Measure	Total number of visits a day	Total visit duration a day (hh:mm:ss)
OSC	Winter	Max	13	3:04:18
		Mean	4.88	00:18:48
		Min	1	0:00:01
		Overall total	351	21:04:19
	Summer	Max	18	3:16:33
		Mean	8	00:47:46
		Min	1	0:00:01
		Overall total	135	14:19:55
TMU	Winter	Max	14	04:10:20
		Mean	5	00:57:18
		Min	1	00:00:01
		Overall total	673	111:30:05
	Summer	Max	20.5	8:52:25
		Mean	5.18	00:57.10
		Min	1	00:00:01
		Overall total	398.5	71:27:52

### Carcass depletion time versus scavenger presence

**Fig. 7:**
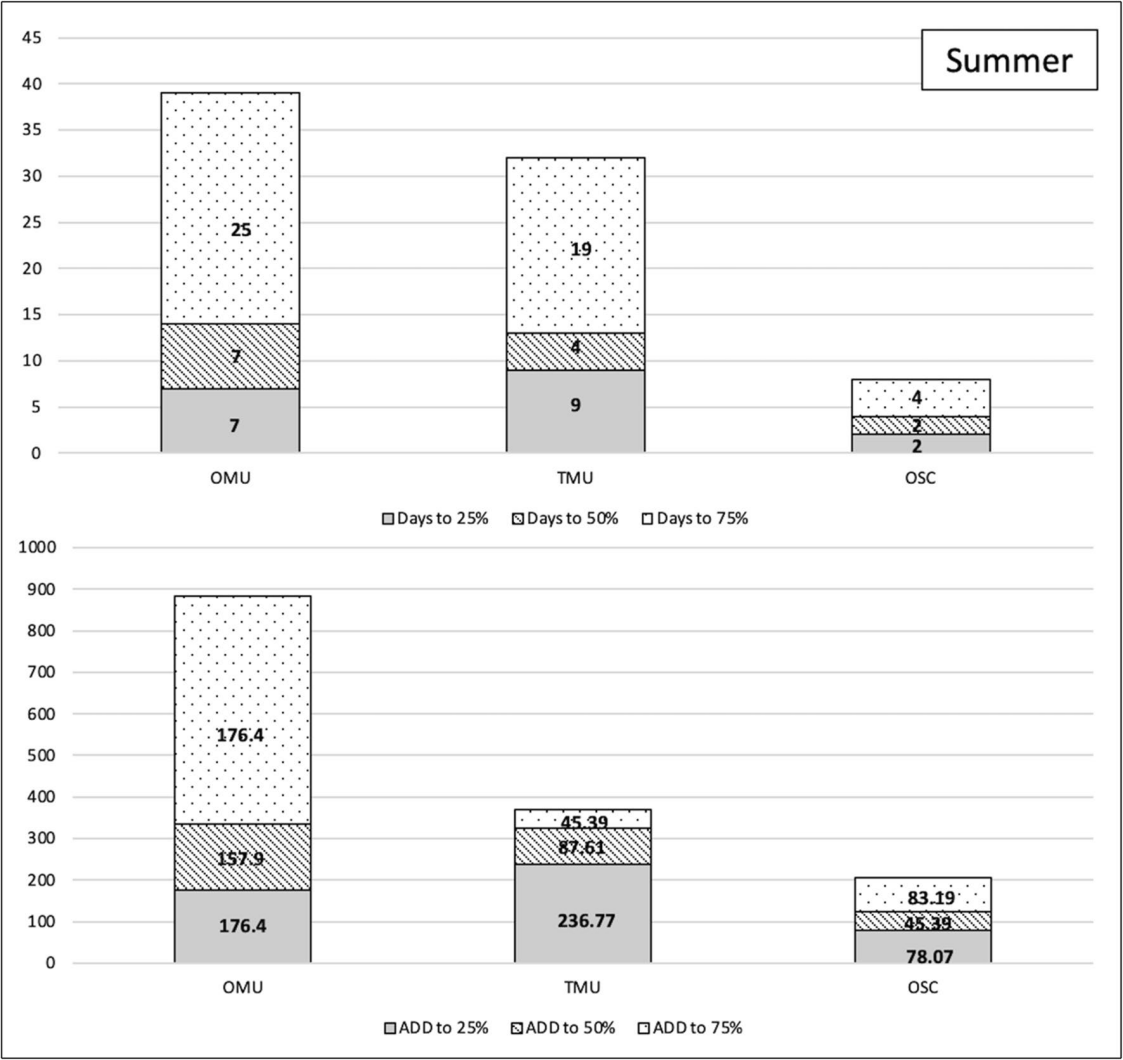
Days and accumulated degree days (ADD) to weight loss milestones—25%, 50%, and 75%—for the summer season of the unclothed thicketed multi-carcass data (TMU) and the unclothed open multi-carcass data (OMU) comparative data and the clothed open single carcass (OSC)

**Fig. 8:**
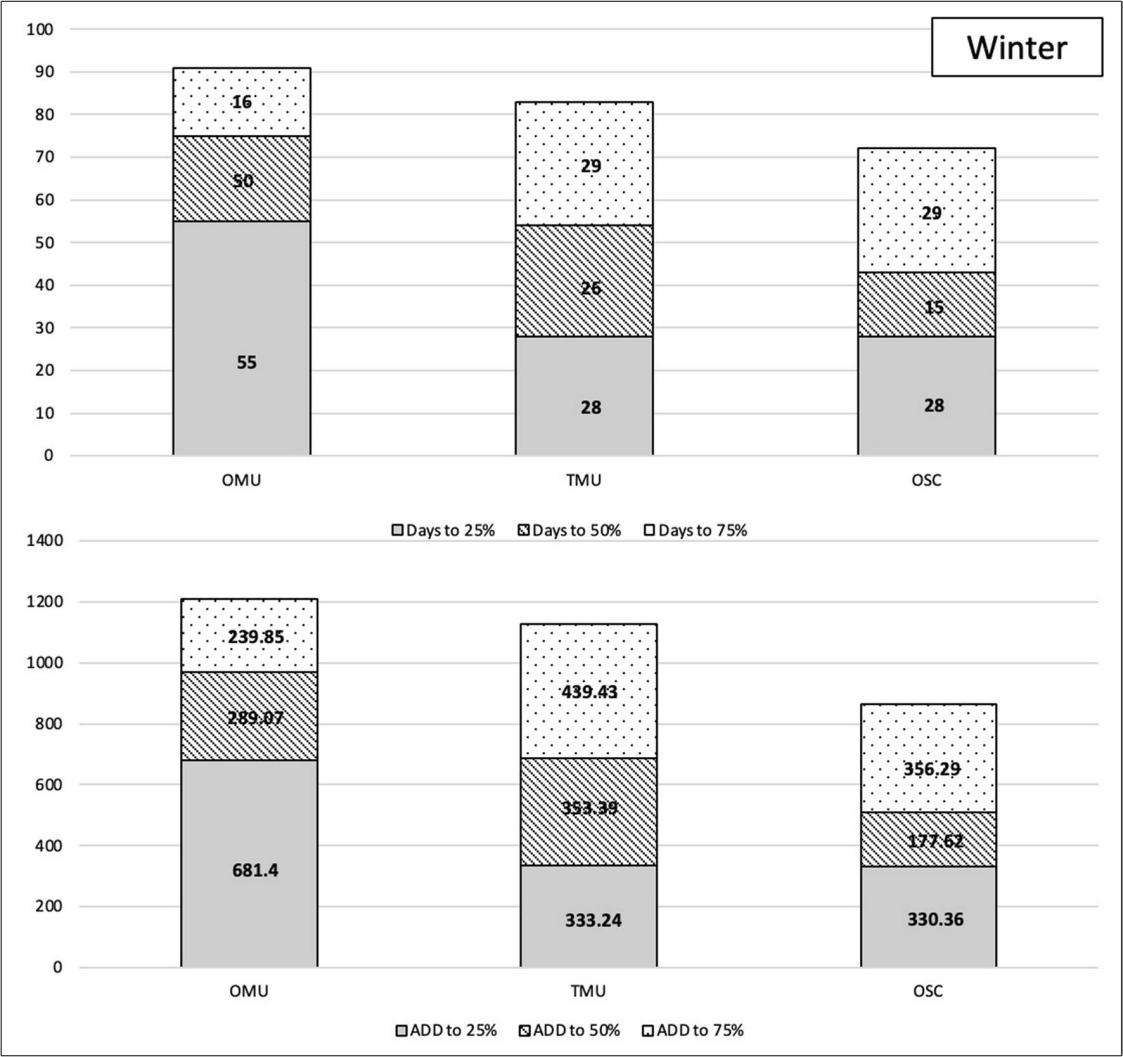
Days and accumulated degree days (ADD) to weight loss milestones—25%, 50%, and 75%—for the summer season of the unclothed thicketed multi-carcass data (TMU) and the unclothed open multi-carcass data (OMU) comparative data and the clothed open single carcass (OSC)

To explore these findings further, we assessed the time (in 24-h days) and accumulated degree days to specific weight loss milestones, namely 25%, 50%, and 75% weight loss. These measures—contrasted by season and carrion biomass load—are presented in Figs. 7 and 8. The results are summarized as follows:

It took less time and accumulated degree days to reach these milestones in summer compared to winter, regardless of habitat or biomass load.

Days to reach these milestones were impacted by the presence of the mongoose scavenging in winter more than summer. The single clothed carcass deployed in the open habitat (OSC), which was scavenged persistently, reached 25% weight loss nearly twice as fast as the carcasses in the unclothed multi-carcass deployment in the same habitat (OMU) that were nearly unscavenged. The time in 24-h days reflects most similarly to the multiple carcasses deployed in the thicketed habitat (TMU), which experienced persistent scavenging. The pattern continues to the 50% milestone, though the difference in time is less pronounced. For the clothed single carcass to reach 75% weight loss, it took more time for the unclothed carcass in the same habitat, illustrating the effect of clothing on decomposition, as scavenging by this point had ceased.

The difference between the open and thicketed multi-carcass deployments was not as pronounced in summer, with the non-scavenged open carcasses progressing quicker through the initial 25% milestone than the thicketed carcasses, highlighting the greater influence of weather over mongoose scavenging in the summer. In summer, the single carcass reached weight loss milestones quicker than all unclothed multi-carcass deployments as it was heavily scavenged and fully exposed to the weather elements.

## Discussion

The impact of clothing and biomass load on scavenging activity was assessed on porcine carcasses in a forensically significant local region of Cape Town, the Cape Flats, where a large proportion of Forensic Anthropology Cape Town (FACT) medico-legal death investigations originate. These results indicate that both clothing and biomass load altered the scavenging behaviour of the Cape grey mongoose and, in turn, the gross decomposition process. During the multi-carcass deployments, the mongoose predominantly fed on the carcasses placed in the thicketed habitat. The absence of the Cape grey mongoose scavenging was observed in the gross carcass decomposition process and specifically the time (in 24-h days) it took for each carcass to reach the weight loss milestones. The small sample size of the OSC deployments is acknowledged, limiting any robust statistical analysis of the results. However, we believe the differences are large enough to warrant close consideration and reporting, with a view to encouraging replication of the experiment both locally and elsewhere for validation of the effects of clothing and carcass biomass load in taphonomic experimental design.

The more similar rate of decomposition in the summer months of both the thicketed and open habitat multi carcass deployments suggests that thermal energy and temperature remains the most significant influence on the decomposition process [4, 5, 39–41]. In summer, differences in temperature between the single carcass deployments and the multi-carcass deployments were found to be significantly different, except for the minimum daytime temperature and maximum nighttime temperature. The average 24-h temperature was 22.8 °C in the OSC deployment, which was 1.6 °C warmer than the mean of multi-carcass deployments. The maximum temperature was greater than the multi-carcass deployments, with a 24-h mean of 42.6 °C while the OSC deployment had a mean of 40.3 °C. The added presence of scavenging on the single carcass further accelerated decomposition process when compared to the multi carcass deployments. However, during the winter, the cooler temperatures meant that the mongoose feeding had a greater impact on the progression of decomposition. In the winter, the OSC carcass decomposed at a similar rate to the TMU carcasses, though these carcasses were still visited more and for longer periods of time than the OSC ones. Both the OSC carcasses and TMU carcasses were scavenged more than the OMU carcasses.

### Scavenging—the effect of clothing and carrion biomass load

The Cape grey mongoose is the predominant scavenger in the CFDS habitat where the experiments were carried out, and the clothing had an influence not only on the scavenging pattern of carcass destruction, but it also, in combination with micro-environment, affected the scavenging activity and in turn the gross carcass depletion time.

The multi-carcass deployments received more mongoose visits and were scavenged for a longer duration compared to single carcass deployments in both seasonal deployments, indicating a scavenger preference for unclothed carcasses. Clothing prevented a large portion of the carcass from being accessed, resulting in less tissue available for consumption. A similar trend was documented by Kjorlien et al. [42], who noted scavenger preference for unclothed carcasses in an open Canadian context, as well as in a Michigan (USA) study by Marshall and colleagues [43] who documented significant differences between their clothed and unclothed caresses regarding the time and abundance of scavenger visits. This research has, therefore, established that the Cape grey mongoose has a definitive preference for the unclothed carcasses regardless of season or interannual effects. Unclothed carcasses provide a larger amount of accessible carrion resource when compared with the clothed carcasses. When the direct amount of time spent scavenging on the remains was compared between OSC carcasses and OMU/TMU carcasses, it was established that the mean average time spent scavenging unclothed carcasses was almost five times more in both the winter and summer season. It should, however, be acknowledged that the difference could also be in part due to the difference of the unclothed carcasses in this situation being situated in the thicketed habitat as opposed to the exposed open habitat, suggesting that protection from predators and/or competitors may influence mongoose scavenging behaviour, especially given this species’ previously documented preference for thicketed habitats [44–46].

The carcasses decomposed much slower in the winter and, as such, the carrion food source was available for a longer period of time, perhaps indicating less of a rush to consume the remains when compared to the summer where the remains were skeletonized within a month. This theory was also used to explain the longer duration of scavenging by racoons in winter [47] and can be extrapolated to the current study. In both seasonal deployments, the mongoose fed predominantly during the fresh and early decomposition stages, although these stages were longer in winter than summer, which led to longer availability of palatable tissue. This trend is also documented by Steadman et al. [48] who found that carrion stays “fresher” in the cooler temperatures and therefore explains why racoons are more active in winter trials. In contrast, Jeong et al. [24] observed racoon scavenging to occur more in summer, but the duration of the racoon visits was greater in winter.

The rapid putrefaction and tissue decay in the summer months may have influenced the feeding as tissue was edible for less time, resulting in rapid feeding. This was also documented by DeVault and Rhodes [49], where 50% of carcasses deployed were removed after 6 days in summer and only 17% of them were removed after 6 days in the cooler temperatures. In the current experiments, the mongoose was documented feeding into the skeletonisation stage during summer, something undocumented in the multi-carcass deployments. It is plausible that clothing in the summer preserved soft tissue for consumption, and perhaps is indicative of lower resource availability in summer pushing the mongoose to select less favourable tissue. This trend was reinforced with the results from Spies [34] who recorded that winter clothed carcasses in the thicketed MRC habitat received more mongoose visits of longer duration compared to summer carcasses, notably, the summer carcasses received longer visits towards the end.

Carrion biomass load greatly alters scavenging activity of the Cape grey mongoose. In winter, the OSC carcass and the TMU carcasses reached the 25% weight loss milestone in the same number of days. This was different from the OMU carcasses, which took 55 days (ADD 681.4) to reach the same milestone This difference can be largely attributed to mongoose scavenging. Both OSC and TMU were heavily scavenged whereas the OMU were not. Another difference was the time (in 24-h days) and ADD it took for all carcasses to reach 50% weight loss: 15 days (ADD 507.98) for the OSC, 26 days (ADD 686.63) for TMU, and 20 days for OMU (ADD 970.47). The differences in this stage may be explained by scavenger swamping, which results in the scavenger dividing their time amongst the various carrion options available, thereby altering the decomposition rate that might be observed if only one carcass was present [21, 50, 51]. This research was reinforced by Du Toit [52, 53] who analyzed the decomposition process of a single clothed carcass in the thicketed CFDS habitat during the winter. The carcass experienced a 400% increase in scavenging activity compared to multi-carcass deployments and reached 75% weight loss in 83 days [52, 53], which is similar to the 72 days it took in the current study. In comparison, Spies and colleagues deployed multiple clothed carcasses and they did not reach the 75% weight loss milestone even after 113 days [33]. The multi-carcass deployments often implemented for statistical robusticity create a surge increasing the carrion biomass load of the habitat, altering the normal scavenging guild of the area [54].

### The importance of experimental design in forensic taphonomy research

This study demonstrates an issue in experimental forensic taphonomic research that we believe should be considered in future research design. The authors acknowledge the current limitations within this project and the disadvantages in the field with the use of using pigs as human proxies [31]. However, this research highlights the impact clothing and carrion biomass can have on scavenging and, as a result, the decomposition process. As most forensic anthropological medico-legal death investigations worldwide consist of clothed individuals [32, 55–57], determining the effect clothing has on the local forensically relevant scavenger species is vital for baseline data studies. Miles et al. [32] also advanced that the shift towards single carcass deployments should be considered during future experimental design in forensic taphonomy research. Multi-carcass deployments are altering the carrion biomass load of a specific environment, creating an artificially large resource compared to what would occur with a single body deposition, thereby altering normal scavenging activity and behaviour. Multi-carcass deployments have been shown here not to provide accurate baseline data for a single carcass deployment. Again, as most forensic anthropological medico-legal death investigations locally include single bodies, this should be accounted for in experimental design. While field experiments have the advantage of realism, research has found that they suffer from low precision due to high between-replicate variation [58]. A retrospective study found that in 160 studies over the course of 30 years (19 for anthropology, 103 for entomology, and 38 for taphonomy), studies in all three disciplines fell victim to pseudo-replication due to the absence of treatment replication, a problem only rectified by conducting more replication experiments [58]. To attain sufficient statistical power from studies within the field of taphonomy, researchers need to ensure proper replication of their research [59]. This involves maintaining spatial independence amongst carcasses, as closely situated carcasses can become interconnected and disperse organisms, potentially leading to the emergence of metacommunities [60]. Additionally, researchers should effectively distribute carcasses to cover a comprehensive spectrum of natural variations, as advocated by Schoenly et al. [58]. Therefore, we recommended multiple single carcass clothed deployments, in the same environment (i.e. within habitat replicates), within the same biogeographic region across multiple seasons and years to adhere to the carrion ecology design rules and provide an accurate validation of research. This methodology is echoed by Schoenly et al. [58] who state that to capture a range of natural variability in post-mortem decomposition, carcasses must be deployed in multiple sites over multiple seasons.

## Conclusion

The aim of this study was to identify the scavenging species of significance in the forensically significant, peri-urban open scrubland habitat in Cape Town, South Africa. The objectives included analyzing and categorizing the scavenging behaviours of animals identified to be interacting with the carcasses and analysing the effect that clothing and biomass load have on scavenging activity across seasons. Scavenging in the peri-urban open habitat in the CFDS habitat was solely by the Cape grey mongoose. The presence of clothing affected scavenging behaviour when compared with unclothed data from the same habitat, resulting in less time spent at the clothed carcasses. Single carcass deployments impacted the scavenging behaviour of the mongoose when compared with the data from the multi-carcass deployments. With the option of multiple carcasses, the mongoose displayed a preference for the protective cover of the thicketed habitat. In contrast, when only one carcass was placed in the exposed CFDS habitat, mongooses had no alternative but to feed in an open setting and did so extensively. Additionally, throughout all seasons, the solitary carcasses underwent faster decomposition and reached significant weight milestones sooner compared to scenarios involving multiple carcasses. The described effects of clothing and carrion biomass load on scavenging behaviours, and in turn the decomposition process assist in the analysis of scavenged remains in South Africa. They also provide insight into the effect the experimental design has on forensic taphonomic research and real-world implications gained from those results.

## Data Availability

The data that support the findings of this study are available from the corresponding author, [VG], upon reasonable request.

## References

[1] Baliso A, Jane Heathfield L, Elaine GV (2023). Informing regional taphonomy research using retrospective forensic anthropology cases in the Western Cape, South Africa. Sci Justice.

[2] Buchan MJ, Anderson GS (2001). Time since death: a review of the current status of methods used in the later postmortem interval. Can Soc Forensic Sci J.

[3] Goff M (2009). Early post-mortem changes and stages of decomposition in exposed cadavers. Exp Appl Acarol.

[4] Campobasso CP, Di Vella G, Introna F (2001). Factors affecting decomposition and Diptera colonization. Forensic Sci Int.

[5] Mann RW, Bass WM, Meadows L (1990). Time since death and decomposition of the human body: variables and observations in case and experimental field studies. J Forensic Sci.

[6] Haglund WD, Reay DT, Swindler DR (1988) Tooth mark artifacts and survival of bones in animal scavenged human skeletons. Journal of Forensic Sciences 33. 10.1520/jfs12521j 3171511

[7] Young A, Stillman R, Smith MJ, Korstjens AH (2015). Applying knowledge of species-typical scavenging behavior to the search and recovery of mammalian skeletal remains. J Forensic Sci.

[8] O’Brien RC, Forbes SL, Meyer J, Dadour IR (2007). A preliminary investigation into the scavenging activity on pig carcasses in Western Australia. Forensic Sci Med Pathol.

[9] Ubelaker DH, DeGaglia CM (2020). The impact of scavenging: perspective from casework in forensic anthropology. Forensic Sci Res.

[10] Willey P, Snyder LM (1989). Canid modification of human remains: implications for time-since-death estimations. J Forensic Sci.

[11] Evert L (2011). Unidentified bodies in forensic pathology practice in South Africa: demographic and medico-legal perspectives.

[12] Steyn M, L’Abbé EN, Myburgh J (2016) Forensic anthropology as practiced in South Africa. In: Blau S, Ubelaker D (eds) Handbook of forensic anthropology and archaeology. London, UK: Routledge; pp 151–164. 10.4324/9781315528939.ch12

[13] Wild S (2017) Three-part journalistic series on South African mortuaries. Available: https://mg.co.za/article/2017-01-12-00-long-quest-to-understand-these-bodies-without-identities/. Accessed 7 January 2024

[14] Reid KM, Martin LJ, Heathfield LJ (2020). Bodies without names: a retrospective review of unidentified decedents at Salt River Mortuary, Cape Town, South Africa, 2010–2017. S Afr Med J.

[15] Reid KM, Martin LJ, Heathfield LJ (2019). Evaluation of DNA profiles obtained from deceased individuals at Salt River Mortuary (South Africa). Aust J Forensic Sci.

[16] Gibbon VE, Finaughty C, Moller I, Finaughty DA (2022) Pressing need for national governmental recognition of forensic anthropology in South Africa as illustrated in a medico-legal case. Sci Justice 411–417. 10.1016/j.scijus.2022.05.003. 10.1016/j.scijus.2022.05.003 35931446

[17] South African Police Service (2021) Crime statistics 2020/2021. Available: https://www.saps.gov.za/services/crimestats.php. Accessed 23 June 2023

[18] Statistics South Africa (2011) Population statistics. Available: https://www.statssa.gov.za/?page_id=964. Accessed 17 June 2023

[19] Finaughty DA (2019) The establishment of baseline data on the rates and processes of soft-tissue decomposition in two terrestrial habitats of the Western Cape, South Africa. PhD thesis. The University of Cape Town, South Africa

[20] Spies MJ, Finaughty DA, Gibbon VE (2018). Forensic taphonomy: scavenger-induced scattering patterns in the temperate southwestern Cape, South Africa — a first look. Forensic Sci Int.

[21] Spies MJ, Gibbon VE, Finaughty DA (2018). Forensic taphonomy: vertebrate scavenging in the temperate southwestern Cape, South Africa. Forensic Sci Int.

[22] Colard T, Delannoy Y, Naji S, Gosset D, Hartnett K, Bécart A (2015). Specific patterns of canine scavenging in indoor settings. J Forensic Sci.

[23] Haglund WD, Haglund WD, Sorg MH (1997). Dogs and coyotes: postmortem involvement with human remains. Forensic taphonomy: the postmortem fate of human remains.

[24] Jeong Y, Jantz LM, Smith J (2015). Investigation into seasonal scavenging patterns of raccoons on human decomposition. J Forensic Sci.

[25] Omond KJ, Winskog C, Cala A, Byard RW (2017). Neonatal limb amputation-an unusual form of postmortem canine predation. J Forensic Sci.

[26] Stevens BS, Reese KP, Connelly JW (2011). Survival and detectability bias of avian fence collision surveys in sagebrush steppe. J Wildl Manag.

[27] Forbes SL, Samson C, Watson CJ (2022). Seasonal impact of scavenger guilds as taphonomic agents in Central and Northern Ontario, Canada. J Forensic Sci.

[28] O’Brien RC, Forbes SL, Meyer J, Dadour I (2010). Forensically significant scavenging guilds in the southwest of Western Australia. Forensic Sci Int.

[29] O’Brien RC, Appleton AJ, Forbes SL (2017). Comparison of taphonomic progression due to the necrophagic activity of geographically disparate scavenging guilds. Can Soc Forensic Sci J.

[30] Rebelo AG, Boucher C, Helme N, Mucina L, Rutherford MC, Smit WJ, Powrie LW, Ellis F, et al (2006) Fynbos biome. In: Mucina L, Rutherford MC (eds) Strelitzia 19: The vegetation of South Africa, Lesotho and Swaziland. Pretoria: South African Biodiversity Institute. 53–219

[31] Matuszewski S, Hall MJ, Moreau G, Schoenly KG, Tarone AM, Villet MH (2020). Pigs vs people: the use of pigs as analogues for humans in forensic entomology and Taphonomy Research. Int J Legal Med.

[32] Miles KL, Finaughty DA, Gibbon VE (2020). A review of experimental design in forensic taphonomy: moving towards forensic realism. Forensic Sci Res.

[33] Spies MJ, Finaughty DA, Friedling LJ, Gibbon VE (2020). The effect of clothing on decomposition and vertebrate scavengers in cooler months of the temperate southwestern Cape, South Africa. Forensic Sci Int.

[34] Spies MJ (2022) The effect of clothing and carrion biomass load on decomposition and scavenging in a forensically significant thicketed habitat in Cape Town, South Africa. PhD thesis. The University of Cape Town, South Africa

[35] Finaughty DA, Pead J, Spies MJ, Gibbon VE (2023). Next generation forensic taphonomy: automation for experimental, field-based research. Forensic Sci Int.

[36] Keough N, Myburgh J, Steyn M (2017). Scoring of decomposition: a proposed amendment to the method when using a pig model for human studies. J Forensic Sci.

[37] Dibner H, Mangca Valdez C, Carter DO (2019). An experiment to characterize the decomposer community associated with carcasses (Sus scrofa domesticus) on Oahu. Hawaii J Forensic Sci.

[38] French GM (2020) Identifying potential sites of distinct scavenger markings left by the Cape grey mongoose (Galerella pulverulenta). MSc thesis. Univesity of Kent, Kent United Kingdom

[39] Galloway A, Birkby WH, Jones AM, Henry TE, Parks BO (1989) Decay rates of human remains in an arid environment. J Forensic Sci 34. 10.1520/jfs12680j 2738563

[40] Gill-King H, Haglund WD, Sorg MH (1997). Chemical and ultrastructural aspects of decomposition. Forensic taphonomy: the postmortem fate of human remains.

[41] Megyesi MS, Nawrocki SP, Haskell NH (2005). Using accumulated degree-days to estimate the postmortem interval from decomposed human remains. J Forensic Sci.

[42] Kjorlien YP, Beattie OB, Peterson AE (2009). Scavenging activity can produce predictable patterns in surface skeletal remains scattering: observations and comments from two experiments. Forensic Sci Int.

[43] Marshall AJ, Simon JR, Watson PL (2009) The effect of clothing on scavenger visits and decomposition. American Academy of Forensic Sciences, Pathology Biology Section – 2009

[44] Cavallini P, Nel JAJ (1990). The feeding ecology of the Cape grey mongoose, Galerella pulverulenta (Wagner 1839) in a coastal area. Afr J Ecol.

[45] Cavallini P, Nel JAJ (1990). Ranging behaviour of the Cape grey mongoose Galerella pulverulenta in a coastal area. J Zool.

[46] Cavallini P, Nel JAJ (1995). Comparative behaviour and ecology of two sympatric mongoose species (Cynictis penicillata and Galerella pulverulenta). J S Afr Vet Assoc.

[47] Smith JK (2015) Racoon scavenging and taphonomic effects on early human decomposition and PMI estimation. MA thesis. The University of Tennessee, Knoxville, USA

[48] Steadman DW, Dautartas A, Kenyhercz MW, Jantz LM, Mundorff A, Vidoli GM (2018). Differential scavenging among porcine, rabbit, and human subjects. J Forensic Sci.

[49] DeVault TL, Rhodes OE, Shivik JA (2003). Scavenging by vertebrates: behavioral, ecological, and evolutionary perspectives on an important energy transfer pathway in terrestrial ecosystems. Oikos.

[50] Ponce C, Alonso JC, Argandoña G, García Fernández A, Carrasco M (2010). Carcass removal by scavengers and search accuracy affect bird mortality estimates at power lines. Anim Conserv.

[51] Smallwood KS (2007). Estimating wind turbine–caused bird mortality. J Wildl Manag.

[52] Du Toit C (2019). Variation in scavenger activity on the Cape Flats, Western Cape, South Africa.

[53] Spies MJ, Finaughty DA, Gibbon VE (2023). Portion size matters: carrion ecology lessons for medicolegal death investigations—a study in Cape Town, South Africa. J Forensic Sci.

[54] Baruzzi C, Mason D, Barton B, Lashley M. 2018. Effects of increasing carrion biomass on food webs. Food Webs 17. 10.1016/j.fooweb.2018.e00096.

[55] Baliso A, Finaughty C, Gibbon VE (2019). Identification of the deceased: use of forensic anthropology at Cape Town’s busiest medico-legal laboratory. Forensic Sci Int Rep.

[56] Baliso A (2020) Identification of the deceased: a retrospective review of forensic anthropology Cape Town casework. MSc thesis. University of Cape Town, Cape Town, South Africa

[57] Komar DA (1998). Decay rates in a cold climate region: a review of cases involving advanced decomposition from the medical examiner’s office in Edmonton, Alberta. J Forensic Sci.

[58] Schoenly KG, Michaud JP, Moreau G (2016) Design and analysis of field studies in carrion ecology. In: Benbow ME, Tomberlin JK, Tarone AM (eds) Carrion ecology, evolution, and their applications. Boca Raton, Fl,: CRC Press. pp 129–149

[59] Dawson BM, Ueland M, Carter DO, McIntyre D, Barton PS (2023). Bridging the gap between decomposition theory and forensic research on postmortem interval. Int J Legal Med.

[60] Leibold MA, McPeek MA (2006). Coexistence of the niche and neutral perspectives in community ecology. Ecology.

